# A giant intra-abdominal right testicular seminoma in a bilateral undescended testicle: a case report

**DOI:** 10.11604/pamj.2023.44.3.37512

**Published:** 2023-01-03

**Authors:** Muhammad Faruk, Muhammad Asykar Palinrungi, Khoirul Kholis, Syakri Syahrir, Syarif Bakri, Abdul Azis

**Affiliations:** 1 Department of Surgery, Faculty of Medicine, Hasanuddin University, Makassar, Indonesia,; 2 Division of Urology, Department of Surgery, Faculty of Medicine, Hasanuddin University, Makassar, South Sulawesi, Indonesia

**Keywords:** Cryptorchidism, intra-abdominal, undescended testicle, testicular cancer, case report

## Abstract

Cryptorchidism is the most common congenital malformation of the male genitourinary tract. An undescended testicle has a 10% chance of developing cancer, with an intra-abdominal testicle having the highest risk. We present a 24-year-old man with a history of bilateral cryptorchidism, complaining of lower abdominal mass for two months. An abdominal computed tomography scan showed an intra-abdominal mass lesion measuring 13 x 9 cm and displacing the bladder caudally. Exploratory laparotomy revealed a right intra-abdominal testicular tumor. A right orchiectomy and left orchidopexy was performed. Histopathological examination revealed a testicular seminoma. The patient was discharged without complications and was referred to the oncology department for chemotherapy and further management. Our findings support the early treatment and close monitoring of cases of cryptorchidism due to the risk of malignancy as well as the necessity of routine scrotal examinations in all males presenting with an abdominal mass.

## Introduction

Cryptorchidism is a common birth anomaly [1,2]. Approximately 10% of undescended testes are located in the abdominal cavity, and an intra-abdominal testis has been reported to have a higher risk of developing testicular cancer than an inguinal testis. While predominantly unilateral, bilateral cryptorchidism can occur though has been rarely reported, with a ratio of unilateral to bilateral cryptorchidism of 4: 1 [2].

The most prevalent cancer of the undescended testis, especially the intra-abdominal testis, is seminoma [3]. The inability of bilateral testicular descent carries a 40-fold relative risk of malignant transformation [1,3]. Here, we report a rare case of giant intra-abdominal right testicular seminoma with bilateral cryptorchidism.

## Patient and observation

**Patient information:** a 24-year-old Asian man presented with a lower abdominal mass for two months. He had no history of severe illness, tumor, or surgery and no familial history of cancer. Both he and his parents had noticed the absence of both testicles in the scrotum since childhood; however, they were unaware of the need for treatment and thus did not seek medical assistance.

**Clinical findings:** upon clinical examination, there was a palpable floating mass in the lower midline abdomen. Scrotal examinations revealed that the bilateral testicle was neither palpable in the scrotum nor in the inguinal region. His vital signs were within normal limits.

**Figure 1:**
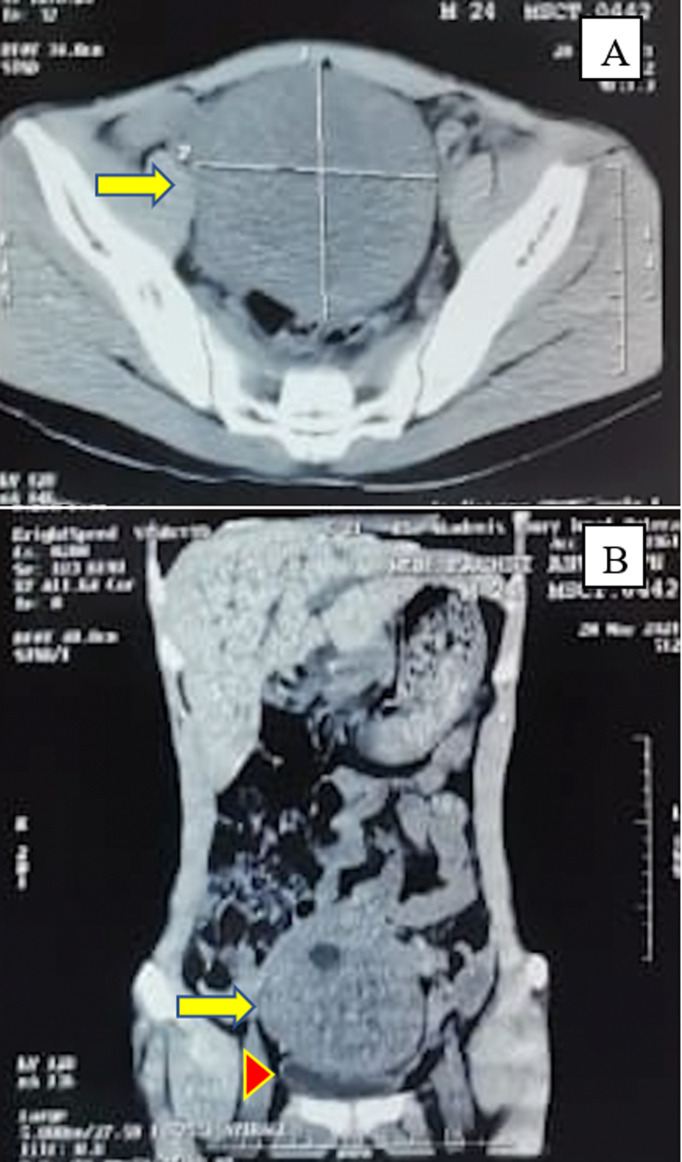
an abdominal computed tomography scan without contrast showing an intra-abdominal mass lesion (yellow arrow) pushing the bladder caudally (red arrowhead) sized 13 x 9 cm; A) axial view; B) coronal view

**Diagnostic assessment:** chest X-rays revealed normal lung fields, and routine blood assessments were within normal limits. Abdominal computed tomography (CT) without contrast showed an intra-abdominal mass lesion measuring 13 x 12 x 9 cm in size and displacing the bladder caudally, with no evident lymph node metastasis or distant metastases (Figure 1). Tumor biomarker assessments, including serum beta-human chorionic gonadotropin (β-HCG), carcinoembryonic antigen, and alpha-fetoprotein, were within normal limits, though serum lactate dehydrogenase was elevated at 2160 U/L (normal range: 140-280 U/L).

**Figure 2:**
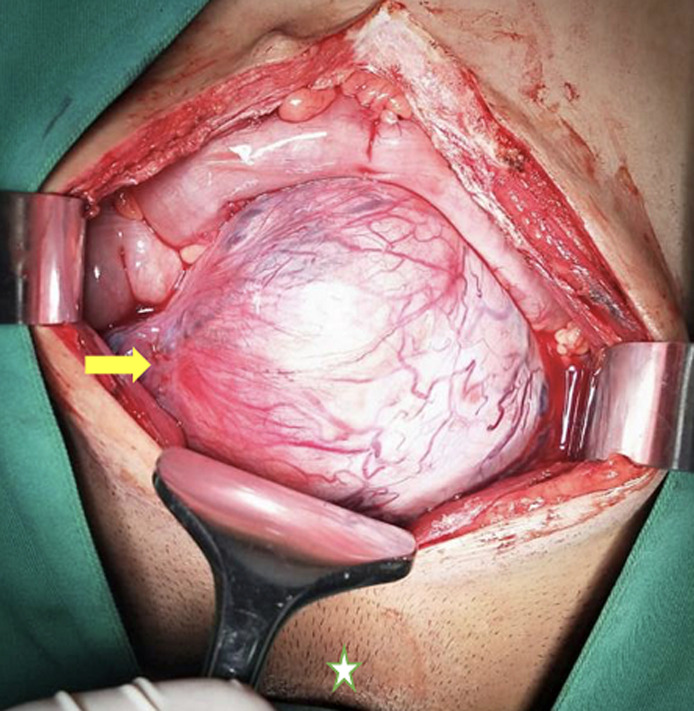
an exploratory laparotomy with a lower midline incision revealing a right intra-abdominal testicular mass (note: star, pubis bone)

**Figure 3:**
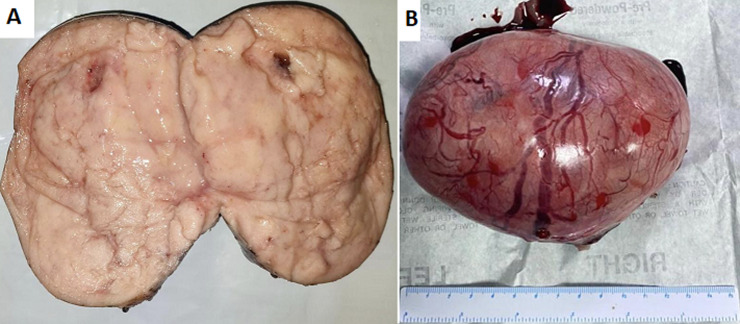
macroscopic features; A) a testicular mass cut surface; B) a testicular mass with a size of 13 x 12 x 9 cm

**Therapeutic interventions:** exploratory laparotomy with a lower midline incision revealed the right intra-abdominal testicular mass (Figure 2). The para-aortic, paracaval, and interaortocaval lymph nodes were not enlarged. During surgery, adhesions were found between the peritoneum and the bowel loops. In addition, fibrous tissue between the small intestines was evident; adhesiolysis was thus performed. An intra-abdominal right orchiectomy was then performed, followed by a left orchidopexy. A testicular mass measuring 13 x 12 x 9 cm in size with a mass of 630 g was removed (Figure 3).

**Figure 4:**
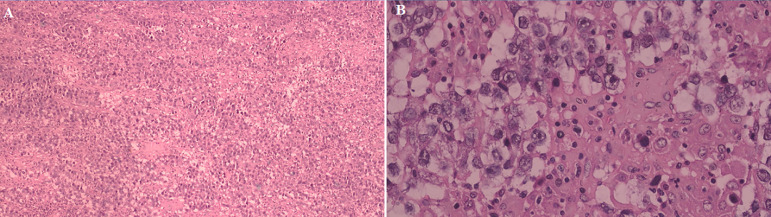
histological examination revealing seminoma of the testicle with A) a sheet-like pattern of clear cells with inflammatory cells and fibrous septae (arrow; H&E staining with 10x magnification) and; B) malignant tumor cells with large round nuclei that were generally monotonous, with coarse nuclear chromatin, prominent nucleoli, and partially clear and partially eosinophilic cytoplasm (arrow; H&E staining with 40x magnification)

Seminoma of the testicle was discovered during the histological examination of the tumor. Neither capsular invasion nor vascularization was present (Figure 4). On laparotomy and CT report, no lymph node metastasis or distant metastases were discovered, and his pathological staging was stage IA (pT1N0M0).

**Follow-up and outcome:** the patient was discharged on the fourth day after surgery and prescribed chemotherapy by the oncology department for further management. Follow-up serum tumor marker evaluation and abdominal CT imaging were conducted four months after surgery with no signs of recurrence.

**Patient perspective:** the patient was pleased with the surgery's positive outcome.

**Informed consent:** written informed consent was obtained from the patient for participation in this study.

## Discussion

Cryptorchidism is characterized by anomalies in testicular development and descent during the embryonic phase. It is found in 6% of full-term newborns and 0.8% of infants under the age of one year. In up to 10% of cases, it is bilateral and sometimes accompanied by other genitourinary tract problems [4].

Cancer is the most feared complication of an undescended testicle, with a risk of 3.5-14.5% among cryptorchidism patients [4-6]. In 10% of cases, the testes are intra-abdominal, putting them at a 200-fold higher risk of malignant transformation [4]. Malignant degeneration is more common in Caucasians and in the third and fourth decades of life [4,7].

They are frequently asymptomatic and are discovered by accident during imaging testing [8]. When symptomatic, the diagnosis might be problematic since symptoms can mirror urinary calculus, mass effects, acute appendicitis, and gastrointestinal and genitourinary tract compressive symptoms [4,9-11].

Ultrasound, CT, and magnetic resonance imaging scans reveal a well-defined, retroperitoneal mass or homogenous pelvic with no apparent calcification or necrosis. These findings have sarcoma and lymphadenopathy as the main differential diagnoses, which are more common circumstances [4].

Surgical treatment is required, including removal of the intra-abdominal mass; however, chemotherapy may be an option depending on the stage and histological type of malignant transformation [4,12]. The reported average size of the abdominal testicular tumors was 13.56 cm, like our patients reported herein [1]. This growth is largely driven by the often absent or nonspecific nature of symptoms. The occurrence of these aggressive late-stage malignancies effectively demonstrates the natural history of uncorrected undescended testes (UDT) and reaffirms our current guidelines for the early correction of cryptorchidism [12].

Hussain reported a case of a man with giant intra-abdominal seminoma presenting with gastrointestinal symptoms of acute right lower abdominal pain who underwent surgery and four cycles of chemotherapy comprising a regimen of bleomycin, etoposide, and cisplatin [13]. Gonda *et al*. reported a case of a man with abdominal pain, vomiting, and hemorrhagic shock due to the rupture of an intra-abdominal testicular seminoma. The patient improved after emergency surgery, though one year postoperatively, the patient had a marked increase in β-HCG, and retroperitoneal lymph node recurrence was found on CT examination. He underwent lymph node dissection and three cycles of chemotherapy with a regimen of bleomycin, etoposide, and cisplatin [9]. Our patient underwent laparotomy with a lower midline incision due to giant testicular seminoma, which was pathologically categorized as stage I. The first line of treatment for stage I testicular seminoma is radical inguinal orchiectomy. Following surgery, standard treatment options include active surveillance, radiation therapy, or one to two carboplatin cycles [9,14]. In our case, serum tumor markers and postoperative abdominal CT imaging were conducted four months after surgery and showed no signs of recurrence. We planned follow-ups every four months for the first year, every six months for the second year, and annually for the third to fifth years.

## Conclusion

Our findings support the early treatment and close monitoring of cases of cryptorchidism due to the risk of malignancy as well as the necessity of routine scrotal examinations in all males presenting with an abdominal mass. Malignancy should be suspected in a patient who has an abdominal mass and undescended testis.
